# Host Range and Emerging and Reemerging Pathogens

**DOI:** 10.3201/eid1112.050997

**Published:** 2005-12

**Authors:** Mark E.J. Woolhouse, Sonya Gowtage-Sequeria

**Affiliations:** * Centre for Infectious Diseases, University of Edinburgh, Edinburgh, United Kingdom

**Keywords:** bacteria, basic reproduction number, epidemiology, fungi, helminths, infectious diseases, protozoa, viruses, reservoir, zoonoses, research

## Abstract

An updated literature survey identified 1,407 recognized species of human pathogen, 58% of which are zoonotic. Of the total, 177 are regarded as emerging or reemerging. Zoonotic pathogens are twice as likely to be in this category as are nonzoonotic pathogens. Emerging and reemerging pathogens are not strongly associated with particular types of nonhuman hosts, but they are most likely to have the broadest host ranges. Emerging and reemerging zoonoses are associated with a wide range of drivers, but changes in land use and agriculture and demographic and societal changes are most commonly cited. However, although zoonotic pathogens do represent the most likely source of emerging and reemerging infectious disease, only a small minority have proved capable of causing major epidemics in the human population.

## Abstract 2

Emerging and reemerging species of human pathogens are associated with a broad range of nonhuman hosts.

A recent, comprehensive literature survey of human pathogens listed >1,400 different species (*1*), more than half known to be zoonotic, i.e., able to infect other host species (*1**,**2*). The survey data showed that those pathogens regarded as emerging and reemerging were more likely to be zoonotic than those that are not (*1**,**3*), confirming an association between these characteristics which had long been suspected (*4**,**5*), but which could not be formally demonstrated without denominator data as well as numerator data.

Here, we revisit these calculations, using updated information on the biology and epidemiology of recognized human pathogens. We pay close attention to possible differences between the major pathogen groups—viruses, bacteria, fungi, protozoa, and helminths. We also examine in detail the relationship between host range and pathogen emergence or reemergence, considering both the type and diversity of nonhuman hosts. We catalog the kinds of proximate factors or drivers that have been linked with pathogen emergence and reemergence and ask whether these differ between the major pathogen groups or between zoonotic and nonzoonotic pathogens.

We focus mainly on pathogen diversity (as numbers of species) rather than on the effects of disease that they impose, noting that many diseases, e.g., infant diarrhea, can be caused by more than one species of pathogen. However, we comment on the transmissibility of pathogens once they have been introduced into the human population because transmissibility is an important determinant of the potential public health problem.

## Methods

We obtained counts of pathogen species from an updated version of the previously published database (*1*). As before, we defined a human pathogen as "a species infectious to and capable of causing disease in humans under natural transmission conditions." We included pathogens that have only been reported as causing a single case of human disease and those that only cause disease in immunocompromised persons. We also included instances of accidental laboratory infection but excluded infections resulting from deliberate exposure in the laboratory. We added recently recognized pathogens listed online by the Centers for Disease Control and Prevention, the World Health Organization (WHO), ProMED, and elsewhere (*6**–**9*). We obtained taxonomic classifications online from the International Committee on Taxonomy of Viruses, the National Centre for Biotechnology Information, the CAB International Bioscience database of fungal names, and from standard texts (*10**–**15*).

Pathogen species were categorized as emerging or reemerging based on previously published reviews of the literature (*1**,**3*), again updated from online sources (*6**–**8*). A species was regarded as emerging or reemerging if any recognized variant fell into this category (e.g., *Escherichia coli* O157, H5N1 influenza A).

We considered the following pathogen groups: viruses (including prions), bacteria (including rickettsia), fungi (including microsporidia), protozoa, and helminths. We did not consider ectoparasites (ticks and lice). Each group was further divided into subgroups (families) to test whether biases existed in numbers of emerging and reemerging species at this level. The viruses were also divided according to genome type (e.g., negative single-stranded RNA viruses).

We examined 3 aspects of host range, both for all pathogens combined and separately for each of the viruses, bacteria, fungi, protozoa, and helminths. First, we distinguished pathogen species according to whether they were known to be zoonotic, using the WHO definition "diseases or infections which are naturally transmitted between vertebrate animals and humans" (*16*). Note that this definition includes pathogens for which humans are the main host and other vertebrates are only occasional hosts, as well as the opposite, but excludes purely human pathogens that recently evolved from nonhuman pathogens, e.g., HIV. We then compared the fraction of emerging or reemerging species that were or were not zoonotic across the major pathogen groups and within each group by family.

Second, for all zoonotic species we identified the types of nonhuman vertebrate host they are known to infect, using the following broad categories: bats, carnivores, primates, rodents, ungulates, and other mammals and nonmammals (including birds, reptiles, amphibians, and fish). We excluded vertebrate intermediate hosts of parasites with complex life cycles. Host types were ranked by the number of zoonotic pathogen species associated with them, and rankings were compared by using Spearman rank correlation coefficient.

Third, we obtained a crude index of the breadth of host range by counting the number of the host types that each pathogen species is known to infect: 0 (i.e., not zoonotic), 1, 2, and 3 or more. We compared the fraction of emerging and reemerging species across these 4 classes.

For the emerging and reemerging pathogen species, we identified the main factors believed to drive their increased incidence, geographic range, or both, by conducting a systematic review of the emerging diseases literature. We allocated these drivers to 1 or more broad categories (Table). Note that although we chose categories that we considered to be useful and informative for our immediate purposes, and which were similar to those listed elsewhere (*5*), this is inevitably a subjective procedure and alternative categorizations may be equally valid. We then ranked the drivers (by number of emerging and reemerging pathogen species associated with each) and compared the ranking of drivers for the major pathogen groups and for zoonotic versus nonzoonotic pathogens.

**Table:** Main categories of drivers associated with emergence and reemergence of human pathogens

Rank*	Driver
1	Changes in land use or agricultural practices
2	Changes in human demographics and society
3	Poor population health (e.g., HIV, malnutrition)
4	Hospitals and medical procedures
5	Pathogen evolution (e.g., antimicrobial drug resistance, increased virulence)
6	Contamination of food sources or water supplies
7	International travel
8	Failure of public health programs
9	International trade
10	Climate change

*Ranked by the number of pathogen species associated with them (most to least).

For the zoonotic species, we distinguished those known to be transmissible between humans, allowing that this might be through an indirect route (e.g., a vector or an intermediate host), from those for which humans can only acquire infection (directly or indirectly) from a nonhuman source. For the transmissible zoonotic species, we further distinguished those that are sufficiently transmissible to cause major epidemics in human populations from those that cause only relatively minor outbreaks. This classification was intended to distinguish between pathogens with *R*_0_>1 in humans from those with *R*_0_<1, where *R*_0_ is the basic reproduction number, i.e., the average number of secondary infections produced by a single primary infection introduced into a large population of previously unexposed hosts. Direct estimates of *R*_0_ are unavailable for most zoonotic pathogens.

Throughout the study, we quantified associations as the relative risk (RR) and tested for statistical significance using a standard χ^2^ test (with correction for small expected values). Although these statistical analyses are susceptible to bias introduced by related species (e.g., several species of hantavirus exist, most of which are zoonotic and many of which are regarded as emerging or reemerging), the analysis at the family level is an indication of the extent of any such bias.

## Results

The survey of human pathogens produced a count of 1,407 human pathogen species, with 177 (13%) species regarded as emerging or reemerging (Appendix). Of all pathogen species, 208 are viruses or prions, including 77 (37%) regarded as emerging or reemerging. For bacteria, the counts were 538 and 54 (10%), respectively; for fungi, 317 and 22 (7%), respectively; for protozoa, 57 and 14 (25%), respectively; and for helminths, 287 and 10 (3%), respectively. These numbers differ slightly from those previously published (*1**,**3*) as a result of adjustments to taxonomies and the discovery of previously unknown pathogen species. Clear differences were found between the pathogen groups (χ^2^_4_ = 154.3, p<<0.001), with viruses greatly overrepresented among emerging and reemerging pathogens and helminths underrepresented.

### Pathogen Taxonomy

More than 20 virus families contain human pathogens, with just 4, the *Bunyaviridae*, *Flaviviridae*, *Togaviridae*, and *Reoviridae*, accounting for more than half of the species affecting humans and, likewise, more than half of the emerging and reemerging species. Overall, no significant difference was found between the 9 largest families (pooling the remainder) in the fraction of species regarded as emerging or reemerging (χ^2^_9_ = 14.9, p = 0.09). Nor were any significant differences found according to genome type, e.g., between RNA and DNA viruses (χ^2^_1_ = 0.77, p = 0.38) or between positive and negative single-stranded RNA viruses (χ^2^_1_ =3.1, p = 0.08).

More than 60 bacteria families contain human pathogens; the enterobacteria and the mycobacteria account for the most species and for the most emerging and reemerging species. Overall, no significant difference was found between the 6 largest families (pooling the remainder) in the fraction of species regarded as emerging or reemerging (χ^2^_6_ = 13.6, p = 0.14). Numbers of species of emerging and reemerging fungi, protozoa, and helminths were too small for meaningful comparisons between families, but no indication was found that emerging and reemerging species are concentrated in any particular taxa.

### Host Range

Of the 1,407 human pathogen species, 816 (58%) are known to be zoonotic. In comparison, of the 177 emerging or reemerging pathogens, 130 (73%) are known to be zoonotic. This corresponds to an RR of 2.0 and confirms the expectation that zoonotic pathogens are disproportionately likely to be associated with emerging and reemerging infectious diseases. This pattern varies somewhat across the different pathogen groups: for bacteria and fungi the association is strongest with RRs of 4.0 and 3.2, respectively; for viruses and protozoa, no obvious association was found, with RRs of 1.2 and 0.9, respectively; and for helminths (which are almost all zoonotic but very rarely emerging or reemerging), RR is 0.3. However, the numbers involved are small (particularly for protozoa and helminths), and these differences were not statistically significant (χ^2^_4_ = 4.03, p = 0.40).

**Figure 1:**
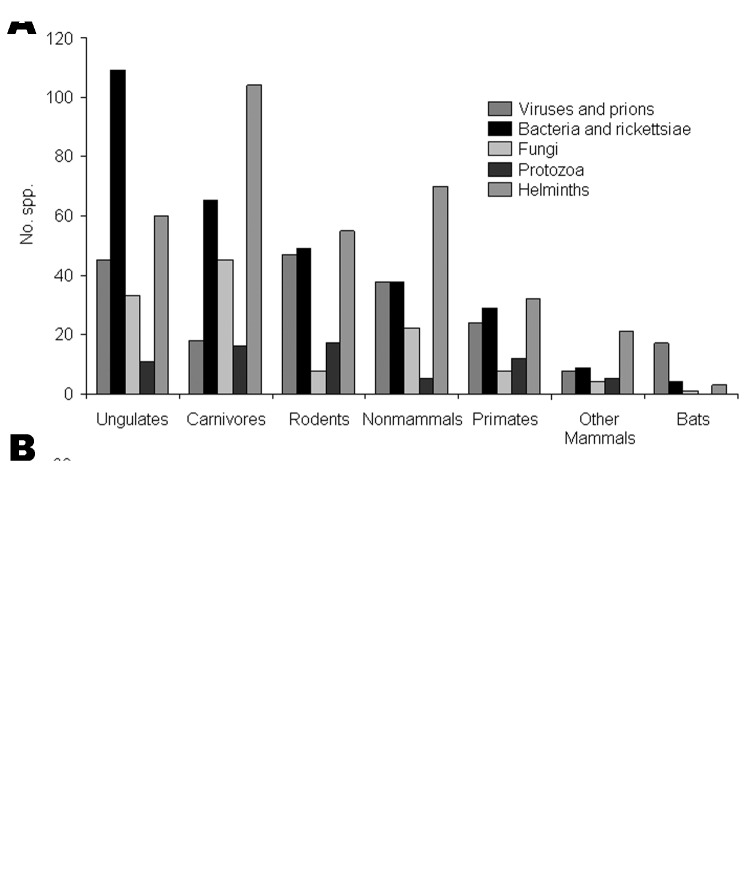
Numbers of species of zoonotic pathogens associated with different types of nonhuman host. Note that some pathogens are associated with >1 host. A) All zoonotic species. B) Emerging and reemerging zoonotic species only.

All the defined host types are potential sources of zoonotic infections, but differences occurred in their importance (ranked by number of pathogen species supported) across viruses, bacteria, fungi, protozoa, and helminths and no 1 type consistently dominates (Figure 1A), although ungulates are the most important overall, supporting over 250 species of human pathogen. Emerging and reemerging pathogens show similar trends (Figure 1B), with ungulates again the most important overall, supporting over 50 species. In general, ranking of host types in terms of numbers of species correlates well both overall (*r_s_* = 0.79, n = 7, p<0.05) and individually for each pathogen group. The general impression is that the emerging and reemerging zoonotic pathogens are not unusual in the types of nonhuman hosts they infect.

**Figure 2:**
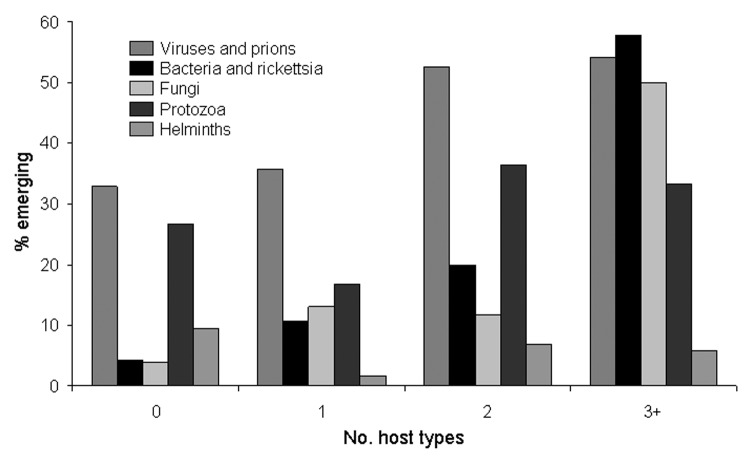
Relationship between breadth of host range (as number of nonhuman host types, as listed in Figure 1) and the fraction of pathogen species regarded as emerging or reemerging. A total of 122 zoonotic species (10 of them emerging or reemerging) for which the host range is unknown are omitted.

However, when the fraction of emerging and reemerging species is compared with the breadth of host range (as the number of host types other than humans), a pattern becomes apparent (Figure 2). Overall, the fraction tends to increase with host range: >40% of pathogens with the broadest host ranges (3 or more types of nonhuman host) are emerging or reemerging (exact p = 0.042). However, this trend does not hold for the protozoa and helminths (although the numbers for these groups are small).

### Drivers of Emergence

We identified 10 main categories of drivers of emergence and reemergence and ranked these by the total number of pathogen species associated with them (Table). The ranking of drivers across different categories of pathogen showed poor concordance (e.g., Spearman rank correlation for bacteria vs. viruses, *r_s_* = 0.41, n = 10, p = 0.24). The most striking discrepancies were as follows: 1) the marked association of emerging or reemerging fungi with hospitalization, poor population health, or both; 2) the greater importance of pathogen evolution and contaminated food and water and the lesser importance of international travel and changes in land use and agriculture for bacteria in comparison with viruses; 3) the greater importance of changing land use and agriculture for zoonoses than for nonzoonoses.

### Transmissibility

Overall, most zoonotic pathogens are either not transmissible (directly or indirectly) between humans at all (i.e., humans are a dead-end host) or are only minimally transmissible. Examples include rabies virus, Rift Valley fever virus, and *Borrelia burgdorferi* (the agent of Lyme disease). A small minority (≈10%) of pathogen species that are technically zoonotic are, in fact, spread almost exclusively from person to person (e.g., *Mycobacterium tuberculosis* or measles virus) or can do so once successfully introduced from a nonhuman source (e.g., some strains of influenza A, *Yersinia pestis*, or severe acute respiratory syndrome (SARS) coronavirus). However, a substantial minority of zoonotic pathogens (about 25%, i.e., 200 species) are capable of some person-to-person transmission but do not persist without repeated reintroductions from a nonhuman reservoir (e.g., *E. coli* O157, *Trypanosoma brucei rhodesiense*, or Ebola virus). This pattern is fairly consistent across the major pathogen groups.

## Discussion

Humans are affected by an impressive diversity of pathogens; 1,407 pathogenic species of viruses, bacteria, fungi, protozoa, and helminths are currently recognized. Of this total, 177 (13%) pathogen species are considered emerging or reemerging. This number must be viewed with some caution, given that these terms are still used somewhat subjectively. More rigorous definitions of emerging and reemerging have been proposed (*5**,**17**,**18*), but these are difficult to apply universally because they require long-term data on distributions and incidences which are available for only a small subset of infectious diseases (e.g., malaria [19] and tuberculosis [20]). Moreover, the counts of emerging and reemerging pathogen species reported here are subject to ascertainment bias. Despite these caveats, our results suggest that pathogens associated with emerging and reemerging diseases share some common features.

First, emerging and reemerging pathogens are disproportionately viruses, although they are not disproportionately different kinds of viruses. Numerically, RNA viruses dominate, comprising 37% of all emerging and reemerging pathogens. RNA viruses are also prominent among the subset of emerging pathogens that have apparently entered the human population only in the past few decades, such as HIV or the SARS coronavirus (*21**,**22*). A possible explanation for this observation is that much higher nucleotide substitution rates for RNA viruses permit more rapid adaptation, greatly increasing the chances of successfully invading a new host population (*21**,**22*).

Second, emerging and reemerging pathogens are not strongly associated with particular nonhuman host types, although emerging and reemerging pathogens more often are those with broad host ranges that often encompass several mammalian orders and even nonmammals. This pattern is consistent across the major pathogen groups. The determinants of host range in general remain poorly understood, but among viruses for which the cell receptor is known, an association exists between host range and whether the receptor is phylogenetically conserved (as measured by the homology of the human and mouse amino acid sequences) (*23*).

Emerging and reemerging pathogens have been likened to weeds (*24*), and that the associations reported above are likely reflecting underlying "weediness," that is, a degree of biologic flexibility that makes certain pathogens adept at taking advantage of new epidemiologic opportunities. This characteristic seems to be reflected in the broad range of drivers of the emergence or reemergence of pathogens, ranging from changes in land use and agriculture, through hospitalization to international travel. Although some drivers are numerically more important than others, the overall impression is that pathogens are exploiting almost any change in human ecology that provides new opportunities for transmission, either between humans or to humans from a nonhuman source.

**Figure 3:**
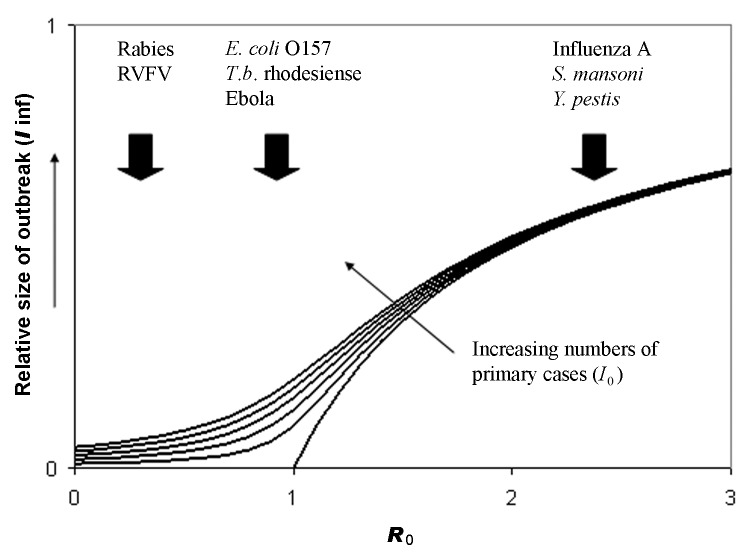
Expected relationship between outbreak size (as fraction of the population affected) and 2 key epidemiologic parameters: I0 is the number of primary cases of infection introduced into the human population from an external source such as a zoonotic reservoir (increasing in the direction indicated); R0 is the basic reproduction number, a measure of the transmissibility of the infection with the human population (see text). The curves are obtained from a modified version of the Kermack-McKendrick equation and show that expected outbreak size is particularly sensitive to small changes in I0 or R0 when R0 is close to 1. Examples of zoonotic pathogens with R0>1, R0<1 and R0 close to 1 are shown. RIVF, Rift Valley fever virus. (Reprinted with permission from [23]).

Even if a pathogen is capable of infecting and causing disease in humans, most zoonotic pathogens are not highly transmissible within human populations and do not cause major epidemics. The possible magnitude of an infectious disease outbreak is related to the basic reproduction number, *R*_0_ (Figure 3). For pathogens that are minimally transmissible within human populations (*R*_0_ close to 0), outbreak size is determined largely by the number of introductions from the reservoir. For pathogens that are highly transmissible within human populations (*R*_0_>>1), outbreak size is determined largely by the size of the susceptible population. For pathogens that are moderately transmissible within human populations (corresponding to *R*_0_ ≈ 1), notable outbreaks are possible (especially if multiple introductions occur), but the scale of these outbreaks is very sensitive to small changes in *R*_0_. In other words, small changes in the nature of the host-pathogen interaction can lead to large increases (or decreases) in the scale of the public health problem (Figure 3). Such pathogens may be likely sources of emerging infectious disease problems in the future. However, we currently have no way of predicting whether a novel human pathogen will behave like rabies (frequently introduced into the human population, but not capable of causing major epidemics) or HIV (probably rarely introduced, but capable of causing a global pandemic).

In conclusion, this study suggests that biologic and epidemiologic correlates of pathogen emergence or reemergence may be identified. However, the most striking feature of emerging and reemerging pathogens is their diversity (Appendix). For this reason, surveillance and monitoring of infectious disease trends may have to be broadly targeted to be most effective. Given that three-fourths of emerging and reemerging pathogens are zoonotic, in many cases this targeting might usefully be extended beyond at-risk human populations to include populations of potential animal reservoirs.

## Appendix

Human pathogen species regarded as emerging or reemerging (5 pathogen groups: viruses and prions; bacteria and rickettsia; fungi; protozoa; and helminths)

### Viruses and prions

Andes

Australian bat lyssavirus

B19

Bagaza

Banna

Barmah Forest

California encephalitis

Cercopithecine herpes

Chikungunya

Crimean-Congo hemorrhagic fever

Dengue

Eastern equine encephalitis

Tickborne encephalitis

Guama

Guanarito

Hantaan

Hendra

Hepatitis A

Hepatitis B

Hepatitis C

Hepatitis E

Hepatitis G

Human astrovirus

Human enterovirus B

Human herpesvirus 1

Human herpesvirus 2

Human herpesvirus 3

Human herpesvirus 5

Human herpesvirus 8

Human immunodeficiency virus 1

Human immunodeficiency virus 2

Human papillomavirus

Human T-lymphotropic virus 1

Human T-lymphotropic virus 2

Influenza A

Japanese encephalitis

Junin

Kyasanur Forest disease

Laguna Negra

Lassa

Machupo

Marburg virus

Mayaro

Measles

Menangle

Monkeypox

Murray Valley encephalitis

Nipah

Norwalk

O'nyong-nyong

Oropouche

Picobirnavirus

Poliovirus

Puumala

Rabies

Reston Ebola

Rift Valley fever

Ross River

Rotavirus A

Rotavirus B

Rotavirus C

Sabia

Salehabad

Sandfly fever Naples

Severe acute respiratory syndrome coronavirus

Seoul

Sin Nombre

Sindbis

St. Louis encephalitis

Venezuelan equine encephalitis

Wesselsbron

West Nile

Western equine encephalitis

Yellow fever

Zaire Ebola

Zika

Bovine spongiform encephalopathy agent

### Bacteria and rickettsia

Aeromonas caviae

A. hydrophila

*A. veronii* (*var. sobria*)

Anaplasma phagocytophila

Bacillus anthracis

Bordetella pertussis

Borrelia burgdorferi

Brucella melitensis

Campylobacter fetus

C. jejuni

Chlamydia trachomatis

Clostridium botulinum

C. difficile

Corynebacterium amycolatum

C. diphtheriae

Ehrlichia chaffeensis

E. ewingii

Enterococcus faecalis

E. faecium

Escherichia coli

Francisella tularensis

Haemophilus ducreyi

H. influenzae

Klebsiella pneumoniae

Legionella pneumophila

Leptospira interrogans

Listeria monocytogenes

Mycobacterium avium

M. bovis

M. fortuitum

M. haemophilum

M. leprae

M. marinum

M. tuberculosis

M. ulcerans

Neisseria gonorrhoeae

N. meningitides

Pseudomonas aeruginosa

Rickettsia prowazekii

Salmonella enteritidis

S. typhi

S. typhimurium

Serratia marcescens

Shigella dysenteriae

Staphylococcus aureus

S. epidermidis

Streptococcus pneumoniae

S. pyogenes

Treponema pallidum

Vibrio cholerae

V. parahaemolyticus

V. vulnificus

Yersinia enterocolitica

Y. pestis

### Fungi

Aspergillus fumigatus

Blastomyces dermatitidis

Candida albicans

Candida glabrata

C. krusei

Coccidioides immitis

Cryptococcus neoformans

Fusarium moniliforme

F. oxysporum

F. solani

Histoplasma capsulatum

Malassezia pachydermatis

Penicillium marneffei

Pneumocystis carinii

Scedosporium prolificans

Trichosporon beigelii

Encephalitozoon cuniculi

E. hellem

E. intestinalis

Enterocytozoon bieneusi

Nosema connori

Trachipleistophora hominis

### Protozoa

Babesia microti

Cryptosporidium hominis

C. parvum

Cyclospora cayetanensis

Giardia duodenalis

Isospora belli

Leishmania donovani

L. infantum

Plasmodium falciparum

P. vivax

Toxoplasma gondii

Trichomonas vaginalis

Trypanosoma brucei

T. cruzi

### Helminths

Anisakis simplex

Echinococcus granulosus

Loa loa

Metorchis conjunctus

Onchocerca volvulus

Schistosoma mansoni

Strongyloides stercoralis

Taenia solium

Trichinella spiralis

Wuchereria bancrofti
